# Immune‐Infiltrated Cancer Spheroid Model with Vascular Recirculation Reveals Temporally Dependent and Tissue‐Specific Macrophage Recruitment

**DOI:** 10.1002/adhm.202402946

**Published:** 2025-02-17

**Authors:** Feng Zhang, Kimia Asadi Jozani, Anushree Chakravarty, Dawn Lin, Andrew Hollinger, Shravanthi Rajasekar, Boyang Zhang

**Affiliations:** ^1^ School of Biomedical Engineering McMaster University Hamilton Ontario L8S 4L8 Canada; ^2^ Department of Chemical Engineering McMaster University Hamilton Ontario L8S 4L8 Canada; ^3^ The Centre for Discovery in Cancer Research McMaster University 1280 Main Street West Hamilton Ontario L8S 4M1 Canada

**Keywords:** circulating immune cells, organ‐on‐a‐chip, tumor microenvironment, vascular perfusion

## Abstract

Immune cell infiltration in tumors has been reported to influence tumor progression and clinical outcomes. Considerable efforts have been made to understand interactions between tumors and the immune system. However, current models are either not comprehensive or limited to short‐term studies. Recognizing thedynamic and long‐term nature of tumor‐immune interactions, an immune‐infiltrated cancer spheroid model is developed by continuously perfusing and recirculating immune cells with gravity‐driven flow through a tubular blood vessel adjacent to a cancer spheroid. Fibroblasts and pericytes are embedded in the gel matrix to support endothelial cells and enhance the vascular barrier. With continuous monocyte recirculation, monocyte adhesion, transendothelium migration, differentiation, and macrophage recruitment into breast carcinoma and hepatoma spheroids is successfully demonstrated over a week. The macrophage recruitment process is temporally dependent and tissue‐specific, leading to the formation of cancer‐macrophage heterospheroids. Elevated secretion of granulocyte‐macrophage colony‐stimulating factor (GM‐CSF), which regulates monocyte recruitment and macrophage activation, is observed in the breast carcinoma model. Increased levels of Interleukin 6 (IL‐6) and Interleukin 8 (IL‐8) are detected, indicating a pro‐inflammatory environment associated with tumor progression and metastasis. This platform provides a valuable framework for investigating immune cell infiltration and differentiation within the tumor microenvironment, supporting the advancement of cancer immunotherapies.

## Graphical abstract

The migration and infiltration of circulating monocytes into cancer spheroids are shown using a pumpless recirculating platform. This method allows for the continuous circulation of immune cells, which is essential for observing the long‐term dynamic behavior of immune cells within cancer environments.

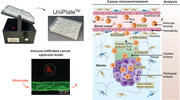

## 1 Introduction

Cancer is the leading cause of death worldwide, accounting for ≈10 million deaths in 2020, or nearly one in six deaths. Cancer immunotherapy has become a focus point of research in the field of tumor immunology over the past decades.^[^ ^1^ ^]^ Our immune system can suppress tumor growth by destroying cancer cells, but it can also promote tumor growth by aiding cancer cells in evading immune surveillance.^[^ ^2^ ^]^ The interactions between cancer and immune systems are complex, making it challenging to devise effective cancer treatment strategies. Modeling these cancer‐immune interactions could be beneficial for better understanding the underlying mechanisms of tumor immunology. In the tumor microenvironment, several factors, such as the endothelium barrier, mural cells, stromal cells, circulating immune cells, and physical blood flow, have been reported to impact tumor progression, angiogenesis, metastasis, and prognostic.^[^ ^3^, ^4^ ^]^ For instance, endothelial cells function as the gatekeeper of immunity, interacting with circulating immune cells in the bloodstream and controlling their extraversion from the blood vessels to the parenchyma by regulating the expression of adhesion proteins such as vascular cell adhesion molecule 1.^[^ ^5^ ^]^ Pericytes have been implicated as mediators of tumor angiogenesis and metastasis, where they are activated and detach from the vessel wall, enabling endothelial cell migration and invasion into the surrounding matrix to form new blood vessels with increased vessel permeability.^[^ ^6^ ^]^ Recently, immune infiltration in tumors has been reported to play a key role in tumor progression and is regarded as a promising target for novel cancer therapeutic strategies.^[^ ^7^, ^8^ ^]^ Therefore, a proper cancer model that integrates those key components of tumor microenvironments is needed to fully recapitulate the physiological conditions of cancer and understand the interactions between the immune system and cancer for clinical cancer therapy.

Many tumor models have been developed, enabling the investigation of tumor progression, angiogenesis, and metastasis processes and mechanisms. These include animal models and organ‐on‐chip tumor models.^[^ ^9^, ^10^ ^]^ Although animal cancer models can maintain the biological characteristics of primary tumors, they fall short in representing the full complexity and functionality of the human immune system, with limited development of human mature innate immune cell populations.^[^ ^11^, ^12^ ^]^ Humanized animal cancer models are time‐consuming and costly to create and maintain, with a relatively short experimental time.^[^ ^13^ ^]^ 3D tumor spheroids models remain a popular approach for exploring tumor responses to anti‐cancer drugs, yet they inadequately represent blood vessel‐tumor and immune‐tumor interactions.^[^ ^14^ ^]^ In contrast, organ‐on‐chip models offer advantages in more precisely controlling the tumor microenvironment and can replicate complex biological processes or interactions, such as tumor‐blood vessel interactions, tumor angiogenesis, tumor‐immune cell interactions, or biochemical gradient.^[^ ^15^, ^16^, ^17^ ^]^ For instance, Meng et al. developed a vascularized tumor model in fibrin gel that demonstrated tumor angiogenesis and tumor metastasis by temporally and spatially manipulating vascular endothelial growth factor (VEGF) or epidermal growth factor (EGF) gradients.^[^ ^18^ ^]^ Neufeld et al. reported a glioblastoma model that mimicked cell diversity and tumor‐stroma interactions in the glioblastoma microenvironment by incorporating pericytes and astrocytes into glioblastoma spheroids and generating blood vessels surrounded by pericytes. This model even exhibited tumor growth curves similar to those in mouse models.^[^ ^19^ ^]^ Recent advancements in tumor modeling have started to integrate immune components into the tumor microenvironment as we now know that the development of a tumor is closely associated with the immune cell recruitment and differentiation process.^[^ ^20^, ^21^, ^22^, ^23^ ^]^ However, current models struggle to replicate this intricate process accurately. A significant hurdle is the continuous recruitment of immune cells from circulation into the cancer microenvironment, a persistent process that contrasts with the standard practice of one‐time cell seeding typically used in tissue production. Current methods mostly use external peristaltic pumps to support long‐term perfusion and recirculation of immune cells.^[^ ^24^, ^25^ ^]^ But the dead volume within the tubing connections and the mechanical stress imposed on cells by these pumps can introduce variability that may compromise cell viability and function. Therefore, there is a pressing need to create a comprehensive tumor model that facilitates the ongoing recruitment of immune cells from the bloodstream into the tumor microenvironment.

**Figure 1:**
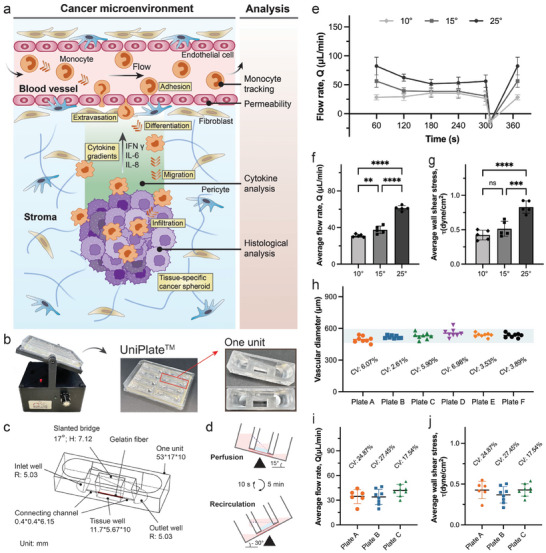
Device setup and flow measurements in UniPlate. a) Schematic overview of cancer microenvironment incorporating circulating monocytes and subsequent analysis protocols in the vascularized cancer spheroid model. b) Image of a programmable rocker and UniPlate^TM^ containing 8 independent units. Each unit is composed of three wells and a slanted bridge that connects the inlet well and the outlet well. c) Dimensions of a single unit within the UniPlate, presented in millimeters (mm). d) Schematic illustration of rocker settings for one perfusion cycle with unidirectional recirculating flow. e) Flow rate changes in the printed vascular channel during one complete perfusion cycle. The flow rate for forward perfusion was quantitatively measured every minute. n = 5 devices for each condition. f, g) Quantification of average flow rate (f) and average wall shear stress (g) in the 5 min of forward perfusion under different tilt angles. n = 5 tissues for each condition. Statistical significance was determined using one‐way ANOVA with the Holm–Sidak method. ^*^ *p* < 0.05, ^**^ *p* < 0.01, ^***^ *p* < 0.001, ^****^ *p* < 0.0001, “ns” indicates not statistically significant. h) Quantification of blood vessel diameters in six different plates. n = 8 for each plate. The coefficient of variation (CV) was determined as the ratio of standard deviation to the mean. i, j) Quantification of average flow rate (i) and average wall shear stress (j) in the 5 min of forward perfusion at the 15° tilt angle. n = 6 tissues for plate A, and n = 8 tissues for plate B and C. The CV was determined as the ratio of standard deviation to the mean.

To recapitulate tumor microenvironments with continuous immune cell circulation (**Figure** **1**a), we report an immune‐infiltrated cancer spheroid model. This model is created by initially seeding endothelial cells in a vascular channel embedded in an extracellular matrix (ECM) hydrogel, then placing cancer spheroids next to the tubular blood vessel, followed by circulating immune cells (monocytes in this work) under flow using a pumpless platform–UniPlate, as we previously published.^[^ ^26^ ^]^ Flow rate characterization confirms that there is a continuous unidirectional flow from the inlet to the outlet well via the blood vessels. Stromal cells, especially fibroblasts and pericytes, which contribute to the barrier function of blood vessels, were included in the cancer spheroid model to reconstitute the interactions between the blood vessels and stroma. Moreover, using the UniPlate, specific monocyte migration and infiltration into cancer spheroids were demonstrated for the first time by continuously recircuiting immune cells using a pumpless recirculating platform. This setup effectively models tissue‐specific monocyte migration and infiltration, showcasing the dynamic process of immune cells responding to cancer. We believe this immune‐infiltrated cancer spheroid model, which recapitulates the interactions among blood vessels, stromal cells, tumors, and circulating immune cells, is a valuable tool for elucidating tumor‐immune interactions.

## 2 Results

### 2.1 UniPlate Setup and Unidirectional Perfusion

The UniPlate devices were manufactured by injection molding and 3D printing, as we have previously described.^[^ ^26^ ^]^ A complete UniPlate consists of three main components: eight units, a printed frame that secures each unit within a designated region, and a one‐well plate for organizing the units (Figure S1a,b, Supporting Information). In this work, we introduced a funnel‐shaped design for the inlet and outlet wells of the UniPlate device (Figure 1b; Figure S1c, Supporting Information). The tissue well is connected to the inlet and outlet wells through a connecting channel measuring 0.4 × 0.4 × 6.15 mm (Figure 1c). This design aims to eliminate dead zones in the corners of wells, thereby enhancing cell recirculation efficiency. The subtractive manufacturing method was used to create perfusable tubular tissues embedded in a hydrogel matrix.^[^ ^27^ ^]^ The printed gelatin template serves as a sacrificial scaffold for the tubular tissues. After removing the gelatin, the open lumen is populated with endothelial cells to produce a tubular blood vessel. For continuous medium perfusion and recirculation, we placed the plate on a programmable rocker. For each perfusion cycle, the rocker first tilts to 15° for 5 min, and then it shifts to the opposite tilt direction and remains at −30° for 10 s to allow the culture medium to flow back (Figure 1d). Flow rate dynamics were analyzed by collecting the medium accumulated in the outlet well over a complete perfusion cycle (5 min of forward perfusion, and 10 s of backflow recirculation). In the gravity‐driven system, the flow rate through the tubular channels decreased progressively during the 5‐min forward perfusion as the culture medium drained from inlet wells to outlet wells but reset at the beginning of each cycle (Figure 1e). The average flow rate increased with higher tilt angles (Figure 1f), resulting in a corresponding increase in the average wall shear stress, which reached 0.92 dyne cm^−2^ at a 25° tilt angle (Figure 1g). However, no significant difference in average wall shear stress was observed between the 10° and 15° tilt angle conditions. Unless otherwise stated, the rocker settings of a 15° tilt angle for 5 min and a −30° tilt angle for 10 s were used throughout this study. A single tubular channel design with an average vascular diameter of ≈530 µm was used in this work. The coefficient of variances (CV) among different plates was between 2.61% and 6.98% (Figure 1h), indicating a high degree of consistency in the dimensions of the engineered tubular channels across different UniPlate devices. We also evaluated the variation of flow conditions among different plates. The average flow rate at a 15° tilt angle was 36.8 ± 4.4 µL min^−1^ (Figure 1i) with an average wall shear stress of 0.41 ± 0.03 dyne cm^−2^ (Figure 1j). Although the shear stress observed in our study is lower than that found in arterial (4–30 dyne cm^−2^) and venous (1–4 dyne cm^−2^) blood flow, it aligns with values reported in prior studies.^[^ ^28^, ^29^, ^30^ ^]^

### 2.2 Stromal Cells Improve Vascular Barrier in 3D Tubular Blood Vessels

**Figure 2:**
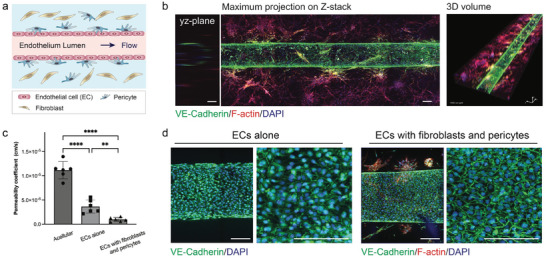
Enhanced barrier function of blood vessels with fibroblasts and pericytes. a) Schematic illustration of vascular tissues containing endothelial cells (ECs), fibroblasts, and pericytes under the continuous recirculating flow. b) Stitched confocal maximum projected z‐stack fluorescence images and 3D‐reconstituted confocal fluorescence image of a blood vessel surrounded by fibroblasts and pericytes under recirculating flow. The cells were stained with VE‐Cadherin (green), F‐actin (red), and DAPI (blue). Scale bar: 200 µm. c) Permeability quantification of acellular vascular channel and vascular tissues formed by endothelial cells alone or endothelial cells with fibroblasts and pericytes. n = 6 tissues for each group. Statistical significance was determined using one‐way ANOVA with the Holm–Sidak method. ^*^ *p* < 0.05, ^**^ *p* < 0.01, ^***^ *p* < 0.001, ^****^ *p* < 0.0001. d) Immunofluorescent staining of blood vessels formed by endothelial cells alone or endothelial cells with fibroblasts and pericytes. The cells were stained with VE‐Cadherin (green), F‐actin (red), and DAPI (blue). Scale bar: 200 µm.

To construct tubular blood vessels in UniPlate, we embedded stromal cells, specifically fibroblasts and pericytes, into a fibrin gel matrix that envelops a tubular channel seeded with endothelial cells (**Figure** **2**a). Under the condition of continuous recirculating flow, the tube developed a contiguous endothelial lining on its inner surface (Figure 2b). The engineered blood vessels, supported by stromal cells, displayed a strong barrier function with a permeability coefficient of 9.75 × 10^−7^ cm^−1^s^−1^, which is comparable to that of rat cerebral microvessels (1.5 × 10^−7^ cm^−1^s^−1^), for 65 KDa dextran.^[^ ^31^ ^]^ Blood vessels with endothelial linings showed significantly lower permeability than the acellular controls, suggesting the function of the endothelial layer in maintaining vascular integrity (Figure 2c). Notably, vessels containing both fibroblasts and pericytes had even lower permeability compared to those lined with endothelial cells alone which showed a permeability coefficient of 3.65 × 10^−6^ cm^−1^s^−1^. This indicates that the presence of stromal cells leads to tighter vascular junctions. The importance of fibroblasts and pericytes in the development and strengthening of blood vessels is supported in previous studies.^[^ ^32^, ^33^ ^]^ The presence of intercellular junctions in vascular tissues, both with and without stromal cells, was further validated by VE‐Cadherin expression (Figure 2d), confirming the establishment of adherens junctions in the engineered blood vessels.

### 2.3 Monocyte Recruitment to Cancer Spheroids is Temporally Dependent

**Figure 3:**
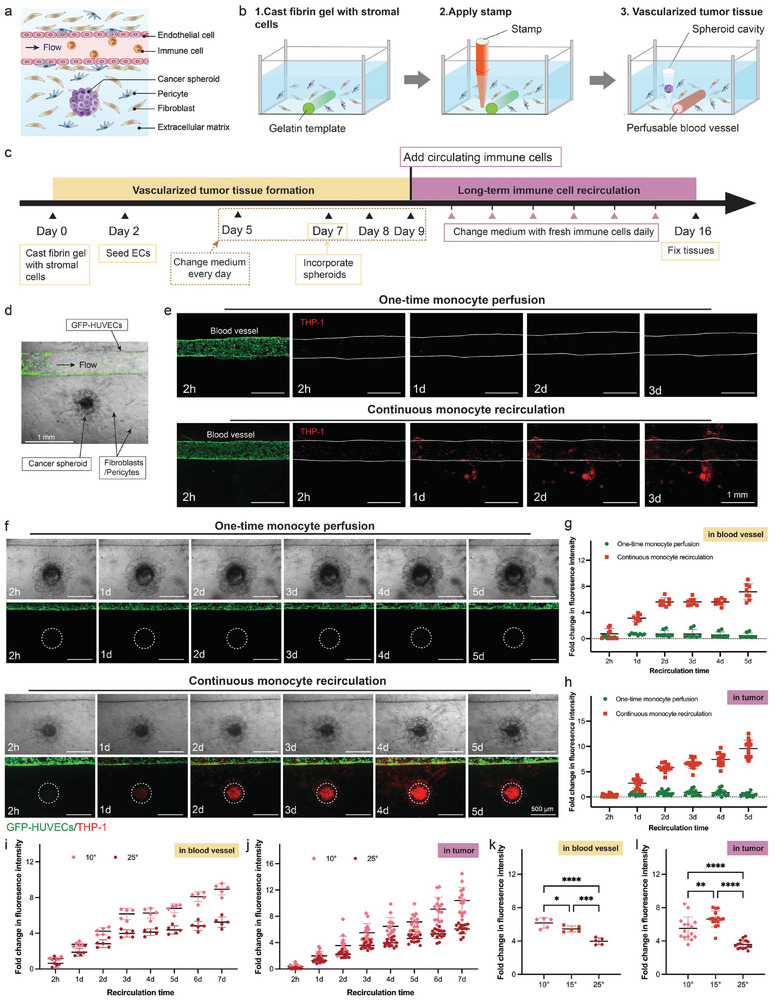
Immune‐infiltrated cancer spheroid model with continuous monocyte recirculation through a blood vessel. a) Schematic illustration of a vascularized cancer spheroid model comprising the blood vessel, stromal cells, and immune components within the tumor microenvironment. b) Schematic illustration of engineering a vascularized tumor tissue using a printed stamp. c) Experimental protocol for long‐term immune cell recirculation in our UniPlate devices, divided into two stages: formation of vascularized tumor tissue and long‐term immune cell recirculation. d) Image of a constructed vascularized tumor tissue with GFP‐HUVECs (green) forming the tubular blood vessel, and fibroblasts and pericytes embedded in the gel matrix. Scale bar: 1 mm. e) Representative images of cell tracker‐labeled monocytes (red) within blood vessels (green) under one‐time monocyte perfusion or continuous monocyte recirculation over time. The edges of blood vessels are outlined with white lines. Scale bar: 1 mm. f) Representative images of red cell tracker‐labeled monocytes (red) at the tumor site under one‐time monocyte perfusion or continuous monocyte recirculation over time. The dashed circle indicates the location of the cancer spheroid. Scale bar: 500 µm. g, h) Quantification of fluorescence signals (red) in blood vessels (g) and at tumor sites (h) with one‐time monocyte perfusion or continuous monocyte recirculation over time. n = 8 tissues were quantified for fluorescence signals in blood vessels, and n = 15 tumor sites were quantified for fluorescence signals in tumors. i, j) Quantification of fluorescence signals (red) within blood vessels (i) and at tumor sites (j) with continuous monocyte recirculation over time under different tilt angles. n = 5 tissues were quantified for fluorescence signals in blood vessels, and n = 15 tumor sites were quantified for fluorescence signals in tumors. k, l) The comparison of fluorescence signals in blood vessels (k) and at tumor sites (l) after 3 days of monocyte recirculation under different tilt angles. n = 5 tissues were quantified for fluorescence signals in blood vessels, and n = 15 tumor sites were quantified for fluorescence signals in tumors. Statistical significance was determined using one‐way ANOVA with the Holm–Sidak method. ^*^ *p* < 0.05, ^**^ *p* < 0.01, ^***^ *p* < 0.001, ^****^ *p* < 0.0001.

Building on the tubular blood vessel, a vascularized tumor model was developed by incorporating cancer spheroids next to the blood vessel using a specially designed printed stamp for precise placement (**Figure** **3**a,b). The stamp was designed with a taper tip (Figure S1d, Supporting Information) and printed with a high‐temperature resin using a FormLab printer, making it suitable for autoclaving and subsequent use in cell culture. Its sharp tip is engineered to penetrate the fibrin gel from above (Figure S1e,f, Supporting Information), leaving behind a cavity that is then filled with cancer spheroids. Our vascularized cancer spheroid model, complete with endothelial lining, stromal support, and immune elements, was established through two phases: initial formation of tumor tissue with its vascular network, and subsequent long‐term recirculation of immune cells (Figure 3c). To visualize immune cell movement, monocytes were marked with a red cell tracker and circulated within the blood vessel.

We explored the impact of sustained immune cell circulation on tumor microenvironments by comparing two scenarios: one‐time monocyte perfusion and continuous monocyte recirculation. In this study, one‐time monocyte perfusion refers to allowing monocytes to pass through the blood vessels a single time during the entire experimental procedure. In contrast, continuous monocyte recirculation involves recirculating immune cells through the blood vessels every 5 min and introducing new monocytes each day. This mimics the average lifespan of human monocytes, which is typically one day.^[^ ^34^ ^]^ In a natural setting, a monocyte circulates through the vascular system multiple times daily, with each loop taking about a minute.^[^ ^35^ ^]^ Our model could theoretically replicate this, allowing for up to 288 circulation cycles in a day, akin to the in vivo frequency. Using the tissue model with MDA‐MB‐231 spheroids as a demonstration, the extent of monocyte adhesion within blood vessels and infiltration into the cancer spheroids was then measured (Figure 3d). The results indicated that continuous monocyte recirculation led to increased monocyte adhesion to the vessel walls over time (Figure 3e). However, the one‐time monocyte perfusion showed minimal monocyte attachment, indicating that continuous monocyte recirculation is necessary for effective immune cell‐tumor interactions. Additionally, a weak fluorescence signal of monocytes was initially detected 2 h after introducing monocytes into the one‐time monocyte perfusion group, but it gradually diminished over time. This result suggests that the interaction between immune cells and tumor blood vessels, after a one‐time perfusion with monocytes, is not stable enough to persist.

To further examine the dynamics of monocyte‐cancer interactions, we monitored the fluorescently labeled monocytes at tumor sites during monocyte perfusion (Figure 3f). A notable increase in the fluorescence signal at tumor sites was observed with continuous monocyte recirculation, indicative of monocyte migration and infiltration into the tumor mass. Conversely, tumor sites subjected to the one‐time monocyte perfusion exhibited no significant increase in the fluorescence intensity, once again demonstrating the importance of continuous monocyte recirculation to effectively study monocyte behaviors and immune responses within tumors. Interestingly, no apparent fluorescence signal was detected at tumor sites following 2 h of continuous monocyte recirculation. However, after one day of sustained monocyte recirculation, the signal became detectable. We quantitatively analyzed the relative fold changes in fluorescence intensity within both blood vessels and at tumor sites to evaluate monocyte adhesion and transmigration (Figure 3g,h). In line with the fluorescence time‐lapse images, the relative change in fluorescence signals within both blood vessels and at tumor sites for the one‐time monocyte perfusion group did not vary significantly over time. In contrast, for the continuous monocyte recirculation group, the fluorescence signal within the blood vessels experienced a 6‐fold increase during the initial three days, stabilizing between days 2 and 4. Moreover, the fluorescence signal within the tumor increased continuously over the 5‐day observation period. This increase suggests that cytokines released by cancer spheroids might be constantly attracting monocytes, prompting their sustained recruitment to the tumor site. In the continuous monocyte recirculation group, we investigated the effect of flow rate on immune cell adhesion and infiltration using the MDA‐MB‐231 cancer spheroid model. To this end, two additional flow conditions with forward perfusion at tilt angles of 10° and 25° were evaluated, and relative fluorescence changes in both blood vessels and tumor sites were quantitatively analyzed. Notably, monocyte viability after 24 h of recirculation at 10° and 25° tilt angles remained comparable to static culture conditions in a standard well plate (Figure S2a, Supporting Information), demonstrating the capability of the UniPlate platform to support immune cell recirculation without compromising viability within this range of shear stress conditions. Primary peripheral blood mononuclear cells (PBMCs) can also be introduced into the UniPlate under recirculating flow conditions. PBMCs viability after 24 h of recirculation was maintained at 78%, demonstrating the feasibility of recirculating immune cells within the UniPlate (Figure S2b,c, Supporting Information). As expected, fluorescence signals within blood vessels and at tumor sites exhibited a persistent increase with recirculation time under both 10° and 25° tilt conditions (Figure 3i,j). After 3 days of continuous monocyte recirculation, fluorescence intensity changes in blood vessels were negatively correlated with the tilt angle, with a 6‐fold fluorescence signal increase observed at 10° compared to a 4‐fold increase at 25° (Figure 3k). Interestingly, fluorescence signal changes at tumor sites were significantly greater at 15° than at 10° or 25° (Figure 3l). These findings suggest that flow rate influences immune cell adhesion and infiltration in our system. Specifically, higher flow rates (25°) resulted in reduced immune cell attachment to blood vessels and diminished immune cell infiltration into tumor sites.

### 2.4 Macrophage Recruitment from Vascular Circulation is Tissue‐Specific

**Figure 4:**
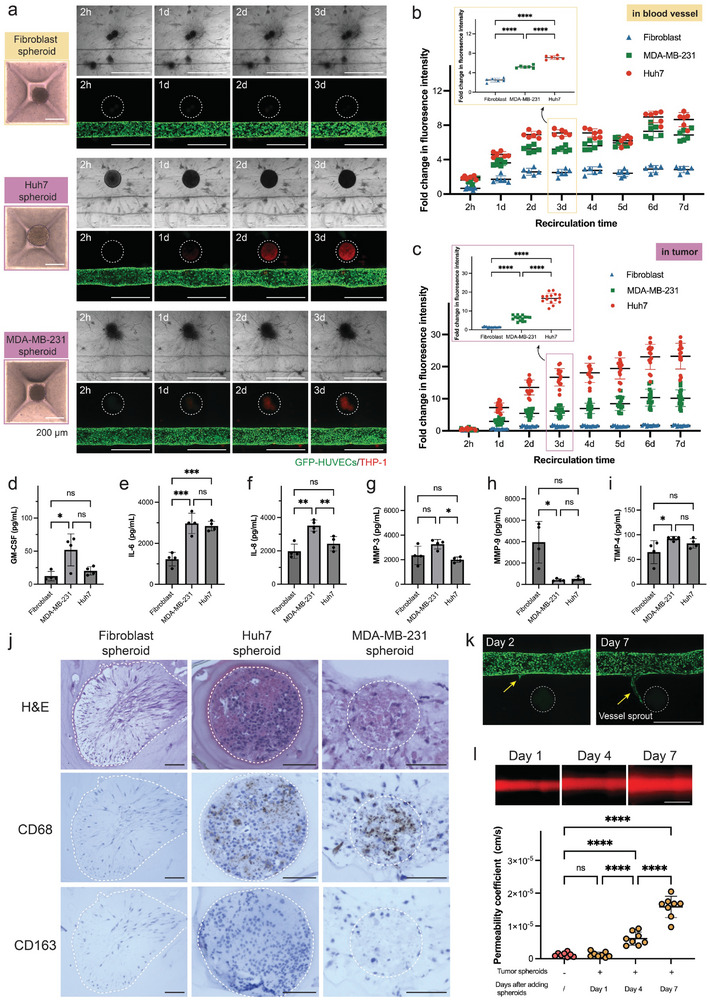
Tissue‐specific macrophage recruitment from the blood vessel to the cancer microenvironment. a) Representative images of fluorescence signals in three types of spheroid models: fibroblast, Huh7, and MDA‐MB‐231 spheroids under continuous monocyte recirculation over time. Red cell tracker‐labeled monocytes were perfused in tissues and replenished daily. The dashed circle indicates the location of the tumor spheroid. Scale bar: 1 mm. b, c) Quantitative analysis of fluorescence signals in blood vessels (b) and in tumors (c) with 7 days of continuous monocyte recirculation. The inset figures show fluorescence signals in three experimental groups after 3 days of monocyte recirculation. n = 6 tissues were quantified for fluorescence signals in blood vessels. n = 15 tumor sites from 6 tissues were quantified for fluorescence signals in tumors. d–f) Secretion levels of representative pro‐inflammatory cytokines, including GM‐CSF (d), IL‐6 (e), and IL‐8 (f) in collected media perfusates from the three tissue‐specific spheroid models. n = 4 tissues for each group. Statistical significance was determined using one‐way ANOVA or Kruskal‐Wallis one‐way ANOVA on ranks with the Holm–Sidak method. ^*^ *p* < 0.05, ^**^ *p* < 0.01, ^***^ *p* < 0.001, ^****^ *p* < 0.0001. “ns” indicates not statistically significant. g–i) Secretion levels of representative MMP cytokines, including MMP‐3 (g), MMP‐9 (h), and TIMP‐4 (i) in collected media perfusates from the three tissue‐specific spheroid models. n = 4 tissues for each group. Statistical significance was determined using one‐way ANOVA or Kruskal‐Wallis one‐way ANOVA on ranks with the Holm–Sidak method. ^*^ *p* < 0.05, ^**^ *p* < 0.01, ^***^ *p* < 0.001, ^****^ *p* < 0.0001. “ns” indicates not statistically significant. j) Histological section of immune‐infiltrated spheroid tissues after 7 days of continuous monocyte recirculation with fibroblast, Huh7, and MDA‐MB‐231 spheroids stained for hematoxylin and eosin (H&E, nuclei stains are blue; ECM and cytoplasm stains are pink), CD68, and CD163 (dark brown). The spheroids are outlined with white dotted circles. Scale bar: 100 µm. k) Vessel sprouting in an MDA‐MB‐231 cancer spheroid model on day 2 (before monocyte perfusion) and day 7 after co‐culturing with cancer spheroids. The cell tracker‐labeled monocytes (red) were introduced on day 2. Scale bar: 1 mm. l) Permeability changes in the blood vessel in the MDA‐MB‐231 cancer spheroid model over co‐culture time. TRITC‐dextran (65 KDa) was perfused through blood vessels for permeability analysis. n = 8 tissues for each group. Statistical significance was determined using one‐way with the Holm–Sidak method. ^*^ *p* < 0.05, ^**^ *p* < 0.01, ^***^ *p* < 0.001, ^****^ *p* < 0.0001. “ns” indicates not statistically significant.

The behavior of monocytes within the tumor microenvironment is multifaceted. Circulating monocytes are activated and drawn to the tumor site in response to a cocktail of inflammatory chemokines, cytokines, and growth factors present within the tumor milieu.^[^ ^36^, ^37^ ^]^ The recruitment process involves endothelium activation, the monocytes adhering to the endothelial layer of blood vessels, then transmigrating across the endothelial barrier into the parenchyma, differentiating into macrophages, and ultimately homing to the tumor location.^[^ ^38^ ^]^ Once situated in the tumor microenvironment, macrophages can further polarize and eventually become tumor‐associated macrophages.^[^ ^39^ ^]^ Different tumor models with immune cell infiltration can be developed using organ‐specific cancer cells. Hepatoma cells (Huh7) were used for modeling liver tumors, and MDA‐MB‐231 cells were selected to model the more aggressive breast tumors, while fibroblast spheroids were prepared as a non‐cancerous control (**Figure** **4**a).^[^ ^40^ ^]^ Compared to the non‐cancerous fibroblast control, both tumor models exhibited significant macrophage signaling just one day after monocyte recirculation, indicating selective monocyte migration into tumor microenvironments. We quantified monocyte/macrophage signals in blood vessels and at tumor sites for fibroblast, Huh7, and MDA‐MB‐231 spheroids (Figure 4b,c). After 3 days of monocyte recirculation, the fluorescence intensity in MDA‐MB‐231 and Huh7 spheroids showed a 5‐ and 7‐fold increase, respectively, significantly higher than that in the fibroblast group.

### 2.5 Cancer Cells Produce Distinct Cytokine Profiles that Promote Macrophage Recruitment

To identify the chemoattractants responsible for monocyte recruitment and migration, medium perfusates after 7 days of continuous monocyte recirculation were collected for cytokine analysis (Figure 4d–g). GM‐CSF, a cytokine involved in monocyte recruitment, macrophage polarization, and inflammatory cytokine production, was secreted significantly more in the MDA‐MB‐231 group, with an upward trend observed in the Huh7 group compared to the non‐cancerous fibroblast control.^[^ ^41^, ^42^ ^]^ This finding correlates with the increased monocyte migration observed in tumor conditions. Tumor models also exhibited higher secretions of inflammatory cytokines IL‐6 and IL‐8, which facilitate monocyte transendothelial migration, in contrast to the non‐cancerous fibroblast control.^[^ ^43^, ^44^ ^]^ The MDA‐MB‐231 group, in particular, displayed significantly more IL‐8 secretion than the Huh7 group. High levels of IL‐8 secretion can support tumor metastasis by enhancing the motility and invasiveness of cancer cells, consistent with the known increased metastatic potential of MDA‐MB‐231 cancer cells and IL‐8′s role in establishing a metastasis niche.^[^ ^45^, ^46^ ^]^ Further analysis of matrix metalloproteinases (MMPs), which impact the ECM degradation and immune cell migration, was conducted (Figure 4h–j). MMP‐3, associated with cancer metastasis and tumor growth in various cancers including breast cancer, showed elevated levels in the MDA‐MB‐231 group vs the Huh7 group, further confirming the aggressive nature of MDA‐MB‐231 cancer cells. No significant difference in MMP‐2 secretion (Figure S3, Supporting Information) was found between non‐cancerous group and cancerous groups, likely due to the complex context of tumor microenvironment where both tumor cells and fibroblasts can secrete MMPs, with levels and activity varying significantly. However, a significantly increased level of tissue inhibitors of metalloproteinases 4 (TMIP‐4), which binds to MMP‐2 to inhibit the degradation of ECM, was observed in the MDA‐MB‐231 group. Lower MMP‐9 levels in the tumor groups compared to the non‐cancerous fibroblast group suggest that fibroblasts might be the primary source of MMP‐9 production in tissues containing fibroblast spheroids.

### 2.6 Assessing Macrophage Polarization and Vascular Responses in Cancer Spheroids

To evaluate macrophage presence and polarization in cancer spheroid models undergoing continuous monocyte recirculation, we performed histological analyses (Figure 4k). Notably, we observed positive CD68 staining, a biomarker for all macrophage types, in both Huh7 and MDA‐MB‐231 spheroids. This confirms the transformation of monocytes into macrophages and their subsequent infiltration into the tumor microenvironment. In contrast, staining for CD163—a marker typically associated with M2‐like macrophages—did not show positive results, suggesting the infiltrated macrophages are likely M1‐type, known for their anti‐tumor activities, which are beneficial during the early stages of cancer progression.^[^ ^47^ ^]^ Histological analysis reveals that CD68(+) macrophages constitute a substantial portion of the overall cell population within the tissue spheroids. However, contrary to expectations, these macrophages have not led to an increase in spheroid size but have instead contributed to increased density over time. Moreover, there is a tendency for these cells to cluster rather than distribute evenly, leading to a spatial pattern reminiscent of the heterogeneity observed in tumor microenvironments.^[^ ^48^ ^]^

In addition to the impact of macrophages on the cancer spheroids, the recruited macrophages could also influence the blood vessels. We occasionally observed vessels sprouting from the pre‐existing vascular tubule toward cancer spheroids in the MDA‐MB‐231 cancer model, a characteristic of tumor microenvironments (Figure 4l). This sprouting occurred before monocyte perfusion and the length of the sprout significantly increased following monocyte recirculation. This increase is likely driven by a VEGF gradient produced by cancer spheroids and infiltrated macrophages, which are known to promote angiogenesis.^[^ ^49^, ^50^ ^]^ Even in the absence of sprouting, we consistently observed a decrease in the barrier function of blood vessels over time in the presence of MDA‐MB‐231 spheroids, similar to what has been reported previously (Figure 4m).^[^ ^51^ ^]^ The expression of VE‐Cadherin, an adherens junction protein involved in regulating vascular permeability and leukocyte transmigration, was detected in the blood vessels of the MDA‐MB‐231 cancer model (Figure S4, Supporting Information). Further characterization using higher‐resolution imaging is required to visualize gaps in the leaky tumor blood vessels.^[^ ^52^ ^]^ The increased permeability of blood vessels could be attributed to the high levels of MMP‐3 secretion in MDA‐MB‐231 models, which disrupts ECM protein and intercellular junction and prepares the blood vessel for angiogenesis.^[^ ^53^ ^]^ Collectively, our immune‐infiltrated tumor models successfully replicate the essential interactions within the tumor microenvironment, particularly the interaction between endothelium, stroma, cancer cells, and immune components.

## 3 Discussion

In this study, we demonstrated that UniPlate can be used to engineer vascularized cancer spheroids with infiltrated immune cells through recirculating flow. The incorporation of pressure‐sensitive adhesives in UniPlate manufacturing significantly reduces the nonspecific absorption of small hydrophobic molecules compared to polydimethylsiloxane surfaces. The absorption profile is comparable to that of standard polystyrene commonly used in conventional multi‐well plates and cell culture flasks.^[^ ^26^ ^]^ The open‐well format of UniPlate enables the easy placement of cancer spheroids to the designated sites using a stamp. This feature allows for the addition of spheroids or organoids at any time point after the formation of blood vessels. We showed the inclusion of fibroblasts and pericytes enhances the barrier function of blood vessels. Furthermore, we achieve long‐term perfusion of monocytes which can circulate through the engineered blood vessels up to 288 times in a day on average, with a daily replenishment of fresh cells, reflecting the natural lifespan and circulation frequency of monocytes in vivo.^[^ ^38^ ^]^ The continuous perfusion and recirculation of immune cells through these vessels leads to the creation of immune‐infiltrated cancer spheroid models that emulate the complex interactions between blood vessels, stromal cells, and circulating immune cells within tumor microenvironments. Currently, the UniPlate platform accommodates eight tissues per plate, but for higher throughput, platforms such as UniPlate96 and UniPlate384 with more compact well designs could be devised. Unlike platforms that rely on pumps, the design of our platform is scalable.

The gravity‐driven mechanism employed in this study generates shear stress levels of up to 1 dyne cm^−2^ within the engineered blood vessels. This shear stress is influenced by multiple factors, including the liquid level difference between the inlet and the outlet wells, the rocker tilt angle, and the dimensions of the engineered tubular channels. Endothelial cells are known to adopt an elongated morphology and align in the direction of flow under unidirectional laminar flow.^[^ ^54^ ^]^ Moreover, exposure to physiological shear stress (≈3 dyne cm^−2^) enhances endothelial integrity by promoting tighter intercellular junctions in the endothelial monolayer.^[^ ^55^ ^]^ In this study, endothelial cell elongation was not observed under the applied shear stress range; however, the incorporation of stromal cells, particularly fibroblasts, and pericytes, into the extracellular matrix improved the permeability of the engineered blood vessels. The impact of shear stress on immune cell responses in the cancer model was also investigated. Under higher shear stress conditions at a 25° tilt angle, immune cell adhesion within blood vessels and infiltration into tumor sites were decreased compared to those observed at 10° and 15° tilt angles. Specifically, immune cell attachment within blood vessels decreased as the tilt angle increased, whereas maximum immune cell infiltration at tumor sites occurred under the 15° tilt condition.

After the addition of cancer spheroids, the blood vessels became inflammatory, showing increased permeability. Immune cells inside these vessels responded to inflammatory signals, exhibiting rolling, adhesion, and transmigration behaviors. High levels of GM‐CSF have been reported to correlate with enhanced cancer metastasis by activating macrophages and stimulating inflammatory cytokine production in mesenchymal breast cancer cells.^[^ ^56^ ^]^ IL‐6 and IL‐8 are considered critical biomarkers for certain cancer types.^[^ ^57^ ^]^ For example, in melanoma patients, elevated levels of IL‐6 and IL‐8 have been linked to decreased median overall survival.^[^ ^58^ ^]^ These cytokines are implicated in the growth, recruitment, and activation of myeloid‐derived suppressor cells, which suppress anti‐tumor immune responses and aid in the tumor's evasion of immune detection. Our study showed a tissue‐specific migration of monocytes toward tumor sites, with an increased secretion of IL‐6 and IL‐8 in cancer tissues vs the non‐cancerous fibroblast control. This selective monocyte trafficking indicates that our immune‐infiltrated cancer spheroid models simulated key features of tumor microenvironments. The monocyte differentiation and macrophage infiltration in tumor microenvironments in our models were confirmed by histological analysis, and the infiltrated macrophages were suggested to be more likely M1 macrophages. M1 macrophages are typically reported to have anti‐tumor effects and are present in the early stage of cancer progression. However, they can be transitioned into M2 macrophages promoting cancer progression and metastasis in tumor microenvironments, owing to the plasticity of macrophages.^[^ ^59^ ^]^ In this study, THP‐1 cells were used to model circulating immune cells. In the future, other immune cells, such as neutrophils, T cells, and PBMCs that are capable of recognizing and eliminating tumor cells can also be included to fully replicate the diverse immune cell population in circulation.

## 4 Conclusions

In summary, we developed immune‐infiltrated cancer spheroid models with long‐term vascular perfusion and continuous recirculation of immune cells using the UniPlate. The incorporation of stromal cells contributes to the engineering of blood vessels with enhanced barrier function, and a vascularized tumor model was developed by integrating tumor spheroids. Through the perfusion and recirculation of immune cells in vascularized tumor tissues, we confirmed that continuous recirculation of immune cells is crucial for the study of immune responses, offering more opportunities for immune cells to traverse blood vessels and achieve effective immune responses. Additionally, tissue‐specific monocyte migration and macrophage infiltration toward tumor sites was demonstrated using three types of spheroids. Overall, our immune‐infiltrated cancer spheroid model with long‐term recirculating vascular perfusion could be used to recapitulate the essential components of the cancer microenvironment for applications in testing immunotherapeutics.

## 5 Experimental Section

### UniPlate Device Manufacturing and Cell Culture

The UniPlate devices (A003, OrganoBiotech, Inc.) were manufactured through injection molding and 3D printing, as previously detailed.^[^ ^26^ ^]^ Each unit consists of two components: a bottomless plate featuring three connecting wells, and an adhesive sheet with printed gelatin templates. Eight units were combined to form a full plate, which was subsequently packaged in a sealing bag for sterilization. Green fluorescent protein‐expressing human umbilical vein endothelial cells (GFP‐HUVECs, Angio‐Proteomie, cat# CAP‐0001GFP) were cultured in Endothelial Cell Growth Medium 2 (ECGM2, Sigma–Aldrich, cat# C‐22011) according to the manufacturer's protocol. Human umbilical vein endothelial cells immobilized with telomerase reverse transcriptase (HUVEC/TERT 2, Evercyte, cat# CHT‐006‐0008) were cultured in ECGM2 medium supplemented with 20 µg mL^−1^ G418 solution (InVivoGen. cat# 108321‐42‐2). Human pericytes from the placenta (hPC‐PL, Sigma–Aldrich, cat# C‐12980) were cultured in pericytes growth medium 2 (Sigma–Aldrich, cat# C‐28041) following the manufacturer's protocol. Human lung fibroblasts were cultured in Dulbecco's Modified Eagle Medium (DMEM, Thermo Fisher Scientific, cat# 11995065) supplemented with 10% fetal bovine serum (FBS, Thermo Fisher Scientific, cat# 12484028), 1% HEPES (1M, Thermo Fisher Scientific, cat# 15630080), and 1% penicillin‐streptomycin glutamine (100X, Thermo Fisher Scientific, cat# 10‐378‐016). Human breast carcinoma cells (MDA‐MB‐231, ATCC), kindly provided by Dr. Fei Geng at McMaster University, were cultured in DMEM supplemented with 10% FBS, 1% penicillin‐streptomycin, and 1% HEPES solution. Human hepatoma cell line (Huh7) cells, a gift from Dr. Shinichiro Ogawa at the University of Toronto, were cultured in DMEM supplemented with 10% FBS, 1% penicillin–streptomycin, and 1% HEPES solution. THP‐1 monocyte cells were cultured in RPMI 1640 medium (Cedarlane Labs, cat# 30–2001) supplemented with 10% FBS and 0.05 mmol L^−1^ 2‐mercaptoethanol (Sigma–Aldrich, cat# M3148‐25ML) in nontreated 6‐well plates. PBMCs were isolated from human whole peripheral blood using SepMate‐50 (IVD) tubes (StemCell technologies). The isolated PBMCs were cryopreserved and stored in a liquid nitrogen tank for future use. For cell passaging, Accutase cell dissociation reagent (Thermo Fisher Scientific, cat# A1110501) was used for detaching pericytes during cell passaging, while 0.05% trypsin‐EDTA (Thermo Fisher Scientific, cat# 25300120) was utilized for all other adherent cells. The endothelial cells, fibroblasts, and pericytes employed in this research were between passages 3 and 7. MDA‐MB‐231 cells were used at passage numbers ranging from 13 to 20. For further experimental preparations, cells were plated in cell culture flasks and grown in an incubator with 5% CO_2_ at 37 °C.

### Spheroids Generation

Commercial Aggrewell 800 plates were used to generate uniform tumor spheroids, following the supplier's instructions. Before cell addition, the Aggrewell plates were treated with an anti‐adherent rinsing solution (Stemcell Technologies, cat# 07010) for 5 min at room temperature. In this work, 0.6 million MDA‐MB‐231 cells and 0.15 million fibroblasts were introduced in a single well of Aggrewell, resulting in each spheroid comprising a total of 2500 cells, including 2000 MDA‐MB‐231 cells and 500 fibroblasts. After centrifuging the cells at 200 rpm for 3 min, the Aggrewell plates were placed in the incubator to allow spheroids formation. Within 2 days of incubation, tightly formed spheroids were achieved and subsequently harvested for further use. Huh7 spheroids were produced following the identical procedure with the same number of cells. Fibroblast spheroids, however, were created in the same manner but solely with fibroblasts. The day after spheroid formation, fresh culture medium was added to the Aggrewells to ensure an adequate nutrient supply. All spheroids from a single well were collected into an Eppendorf tube, and after allowing 5 min for static settlement, the supernatant was removed. The spheroids were then washed once with the fresh medium before being resuspended in 200 µL of a fibrinogen solution (10 mg mL^−1^).

### Vascular Tissue and Vascularized Tumor Tissue Culture

The development of vascular tissues followed the protocol from the previous work, with the modification of including stromal cells within the gel. Fibroblasts and pericytes were mixed in equal parts and resuspended in 10 mg mL^−1^ fibrinogen solution (Sigma–Aldrich, Cat# F3879‐1G) achieving a final density of 0.05 million cells/mL. To prepare the fibrin gel, 100 µL of the fibrinogen solution containing stromal cells was mixed with 20 µL of thrombin solution (10 U mL^−1^ Sigma–Aldrich Cat#, T6884‐100UN). The mixture was promptly cast into one of the tissue wells, ensuring the printed gelatin template was fully enclosed within the gel. Following overnight incubation at 37 °C, an open lumen was created inside the hydrogel by aspirating the gelatin, which had dissolved in PBS, and then the hydrogel was rinsed with a complete medium. Endothelial cells were subsequently seeded into both inlet and outlet wells by adding 50 µL of cell suspension at a density of 2 million cells/mL to form vascular tissues.

For the construction of vascularized cancer spheroid models, a stamp was employed to create cavities atop the gel for cancer spheroids. The stamp, designed as a cylinder with a 600 µm diameter and a sharp tip measuring 200 µm in diameter, was fabricated using a Formlab 3B printer with high‐temperature resin at a 25 µm printing resolution. The utilization of high‐temperature resin enables the sterilization of the stamps through autoclaving. The stamp was inserted into the gel 15 min after the fibrin gel was cast. The stamp remained in the tissue well for an additional 20 min to allow further gelation. The gap between the stamp tip and the bottom of the well was precisely set to 40 µm to address the issue of delamination at close distances and the challenges of monocyte migration at larger distances. To avoid hydrogel adhesion to the stamp, the stamps were treated with an anti‐adherent rinsing solution for 1 h and then air‐dried for 30 min. Upon removing the stamp, cavities for the spheroids were successfully created.

After the formation of confluent blood vessels, the collected spheroids were introduced to the tissues. A fibrin gel, prepared by mixing fibrinogen (10 mg mL^−1^) with the spheroids and thrombin (1 U mL^−1^), was layered over the vascular tissue. Immediately following this addition, the plate was centrifuged at 300 rpm for 10 s to ensure the spheroids settle into the pre‐formed spheroid cavities. The gel was then allowed to be set at room temperature for 20 min, after which a fresh medium containing 1% (v/v) aprotinin (Sigma–Aldrich, Cat# 616370‐100MG‐M) was added to the plate. The plate was subsequently placed on the rocker for culturing. To inhibit the degradation of the fibrin gel over time, all cell culture media were supplemented with 1% (v/v) aprotinin. The ECGM2 containing 1% (v/v) aprotinin was used for the culture of all vascular and vascularized tumor tissues, and the medium for all tissues was refreshed daily.

### Flow Rate Measurements

To quantify the flow rate in tubular channels, the volume of liquid flowing through the tubular channel to the outlet well over a specified period was collected and measured. Initially, 450 µL of phosphate‐buffered saline (PBS) solution containing 1% (v/v) aprotinin was added to the inlet well, and 200 µL was added to the tissue well. The plate was then positioned on a rocker set to 10°, 15°, or 25° tilt angle. The liquid accumulated in the outlet well after intervals of 30, 60, 90, 120, 150, 180, 210, 240, 270, and 300 s during the forward perfusion was collected separately. The backflow rate during the recirculation step was quantified by collecting the liquid flowing from the outlet wells to the inlet wells through the blood vessels over a 10‐s interval. The dynamic flow rate was then measured at 1‐min intervals.

### Permeability Characterization

To evaluate the permeability of engineered blood vessels, the vessels with TRITC‐labeled dextran (65 kDa, Sigma–Aldrich, cat# T1162) was perfused. 200 µL of culture media was introduced into the tissue well and 500 µL of a TRITC‐labeled dextran (1 mg mL^−1^) solution into the inlet well. To evaluate the barrier function of engineered blood vessels, vascular tissues were selected at day 7 post‐endothelial cell seeding, ensuring the establishment of complete endothelial monolayers. To assess permeability changes in the vascularized cancer spheroid model, blood vessels were perfused with the TRITC‐dextran solution on days 1, 3, and 7 following co‐culture with MDA‐MB‐231 cancer spheroids. To visualize the perfusion of the dextran molecule through blood vessels, a microplate reader (BioTek, Cytation 5) was used to capture time‐lapse perfusion images at 30‐min intervals. Subsequently, the diffusive permeability *P_d_* for each blood vessel was calculated using a formula previously reported in the literature.^[^ ^60^ ^]^ In this formula, *I_f_* and *I_i_* denote the average fluorescence intensities at the final and initial time points, while *I_b_* is the average background fluorescence intensity. Δ*t* is the time interval used in the experiment, and *d* is the average diameter of the vessel channel.

(1) $$P_{d}=\frac{1}{\Delta t}\;\times \left(\frac{I_{f}-I_{i}}{I_{i}-I_{b}}\right)\times \frac{d}{4}$$

### Long‐Term Monocyte Recirculation

To demonstrate long‐term perfusion and recirculation of immune cells using the UniPlate, 0.4 million human monocytes (THP‐1) were introduced into the inlet well for each tissue. Before their addition, THP‐1 cells were labeled with the cell tracker red CMTPX (Invitrogen, cat#C34552) following the supplier's instructions. Typically, THP‐1 cells were incubated with a 10 µm dye solution for 30 min in the incubator, followed by a wash with PBS. Then, the labeled monocytes were resuspended in a fresh medium and incubated for an additional 5 h before their introduction to the tissues. For the one‐time monocyte perfusion condition, the plate was left undisturbed for 1 h after adding the monocytes. Then, the cell suspension in both inlet and outlet wells was aspirated and replaced with fresh medium. Afterward, the plate was moved to the rocker for subsequent culturing, with daily medium changes for the tissues. Conversely, for continuous monocyte perfusion and recirculation, the plates were maintained on the rocker throughout the experiment. Tissues were supplied with fresh cell tracker‐labeled monocytes daily during the medium change.

### Cell Viability

To assess the feasibility of recirculating immune cells in UniPlate, immune cell viability was analyzed. THP‐1 cells and PBMCs were introduced into the UniPlate under recirculating flow conditions. Cell viability was quantified after 24 h of recirculation at various tilt angles. Immune cells cultured in a 24‐well plate with magnetic stirring served as the control.

### Immunostaining

All tissues were fixed using a 4% (w/v) paraformaldehyde solution for 1 h at room temperature. Next, the tissues were washed three times with PBS buffer and blocked with a blocking solution (10% FBS in PBS) containing 0.1% Triton X‐100 for 1 h at room temperature. After removal of the blocking buffer, tissues were incubated overnight at 4 °C with the primary antibody, anti‐VE‐Cadherin (Abcam, cat# ab33168), on a rocker. To ensure the complete removal of all residual primary antibodies, the tissues were washed with PBS over 2 days with the PBS refreshed daily. Subsequently, the tissues were incubated for 1 h at room temperature with fluorochrome‐conjugated secondary antibodies, F‐actin (Cedarlane Labs, cat# 20553‐300), and DAPI (Sigma–Aldrich, cat# D9542). Both primary and secondary antibodies were diluted in PBS containing 2% FBS. F‐actin was diluted at a 1:1000 ratio in a 2% FBS solution, and DAPI was diluted at a 1:100 ratio in a 2% FBS solution. Before imaging with a confocal microscope (ZEISS, 3i Marianas LightSheet), the tissues underwent another 1–2 days of washing with PBS.

For histological analysis, tissue samples after 7 days of continuous monocyte recirculation were fixed in a 10% formalin solution for 48 h before being stained and embedded in paraffin wax. Hematoxylin and eosin (H&E) staining was performed, and the selected slides were further stained with CD68 (Agilent, cat# M087601‐2) and CD163 (Abcam, cat# ab182422) antibodies.

### Cytokine Analysis

Cytokine analysis was conducted by Eve Technologies using two specific panels: Human Cytokine Array Proinflammatory Focused 15‐plex panel (cat# HDF15) and Human MMPTIMP 13‐Plex Discovery Assay (cat# HMMP/TIMP‐C,0). Medium perfusates collected from tissues containing fibroblast, MDA‐MB‐231, and Huh7 spheroids after 7 days of continuous monocyte recirculation were prepared for cytokine analysis. The collected medium was centrifuged at 1000 G for 10 min at 4 °C to separate the supernatants, which were then utilized for the cytokine analysis.

### Data Quantification and Statistical Analysis

The fold change in fluorescence intensity in blood vessels and at tumor sites was calculated by analyzing the histogram intensity of fluorescence within the selected regions of interest (ROI) for time‐lapse images using ImageJ software. For statistical analysis, Prism 9 (GraphPad, USA) was employed to test the normality and equality of variance. Depending on these tests' outcomes, either One‐way ANOVA or Kruskal–Wallis one‐way ANOVA on ranks, combined with the Holm–Sidak method at a significant level of p < 0.05, was used to determine statistical significance. Data are presented in all graphs as scatter plots with standard deviation (SD) using Prism 9. To ensure robust quantitative analysis, at least three independent samples (n ≥ 3) were used for each experimental condition.

## Conflict of Interest

The authors declare no conflict of interest.

## Author Contributions

F.Z. carried out the experiments, analyzed the data, and drafted the manuscript. K.A.J. contributed to preparing histology samples and capturing images of the stained samples. A.C. contributed to the injection molding manufacturing of UniPlate devices. D.L. contributed to the refining of immunostaining protocol and carried out confocal imaging of blood vessel tissues. A.H. designed the original stamps for spheroids incorporation and optimized printing files of gelatin templates. S.R. contributed to cytokines sample preparation and results analysis. B.Z. envisioned the concept, supervised the work, and edited the manuscript.

## Supporting information



Supporting Information

## Data Availability

The data that support the findings of this study are available in the supplementary material of this article.
