# Supplementary material for: Immune‐Infiltrated Cancer Spheroid Model with Vascular Recirculation Reveals Temporally Dependent and Tissue‐Specific Macrophage Recruitment
Source: Adv Healthc Mater. 2025 Feb 17;14(9):2402946. doi: 10.1002/adhm.202402946 (PMC11973944; doi:10.1002/adhm.202402946)
Supplement: Supplementary file 1 — Supporting Information [file ADHM-14-0-s001.docx]

**Supporting Information**

**Immune-infiltrated cancer spheroid model with vascular recirculation reveals temporally dependent and tissue-specific macrophage recruitment**

*Feng Zhang^1^, Kimia Asadi Jozani^1^, Anushree Chakravarty^2^, Dawn Lin^2^, Andrew Hollinger^1^, Shravanthi Rajasekar^2^, Boyang Zhang^1,2^**

^1^ School of Biomedical Engineering, McMaster University, Hamilton, Ontario L8S 4L8, Canada;

^2^ Department of Chemical Engineering, McMaster University, Hamilton, Ontario L8S 4L8, Canada;

* Corresponding author, orcid.org/0000- 0002-2060-5555; Email: [zhangb97@mcmaster.ca](mailto:zhangb97@mcmaster.ca)


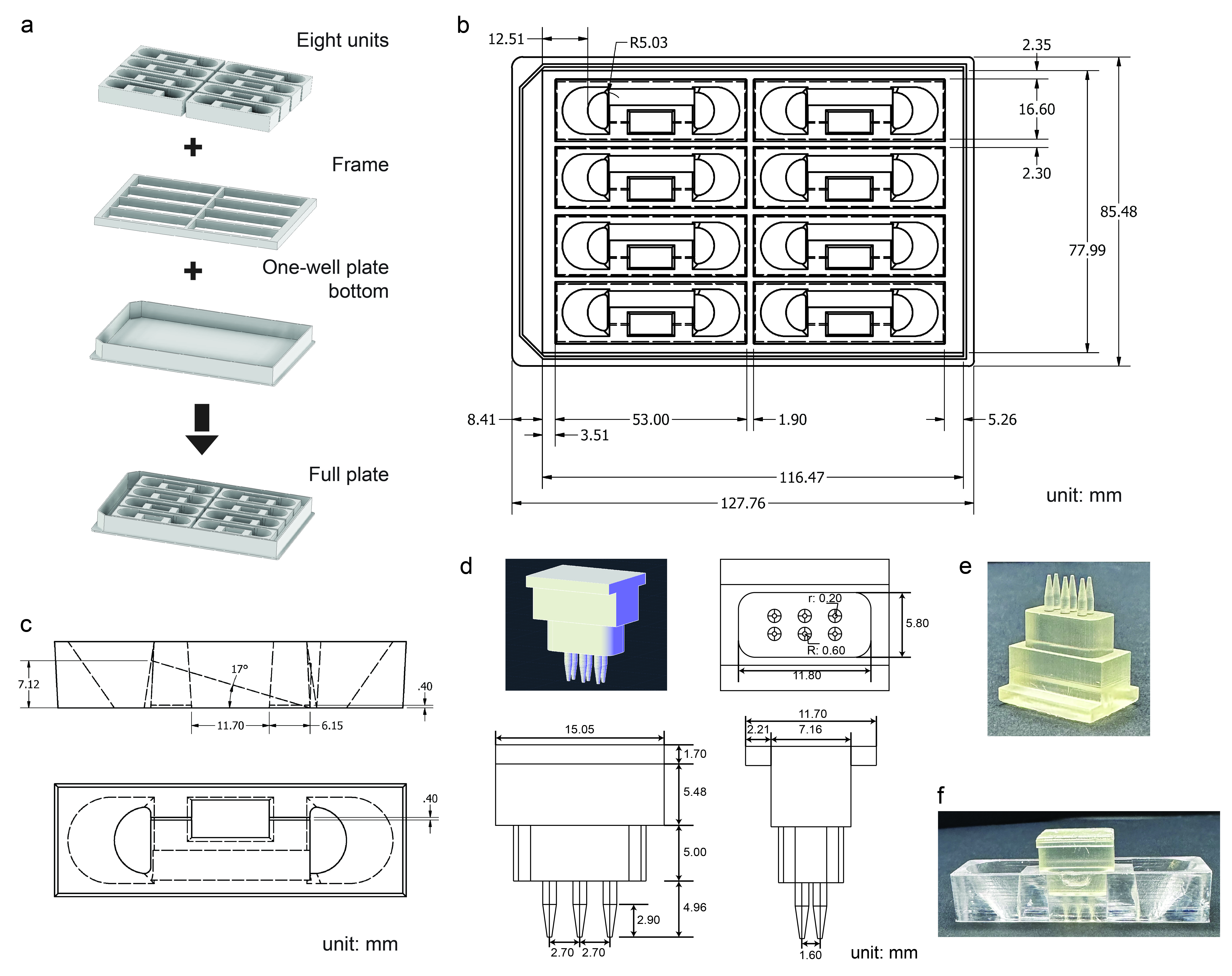


**Figure S1**. Design and dimensions of UniPlate and printed stamp for spheroid incorporation. a, b) The layout (a) and the dimension file (b) of a full UniPlate. c) The dimension of a single unit of UniPlate. d) The design and dimension of the stamp for incorporating spheroids. e) Image of a printed stamp made with high temperature resin. f) Image of a stamp applied to the plate unit. The stamp matches perfectly with the tissue well of the UniPlate.

**
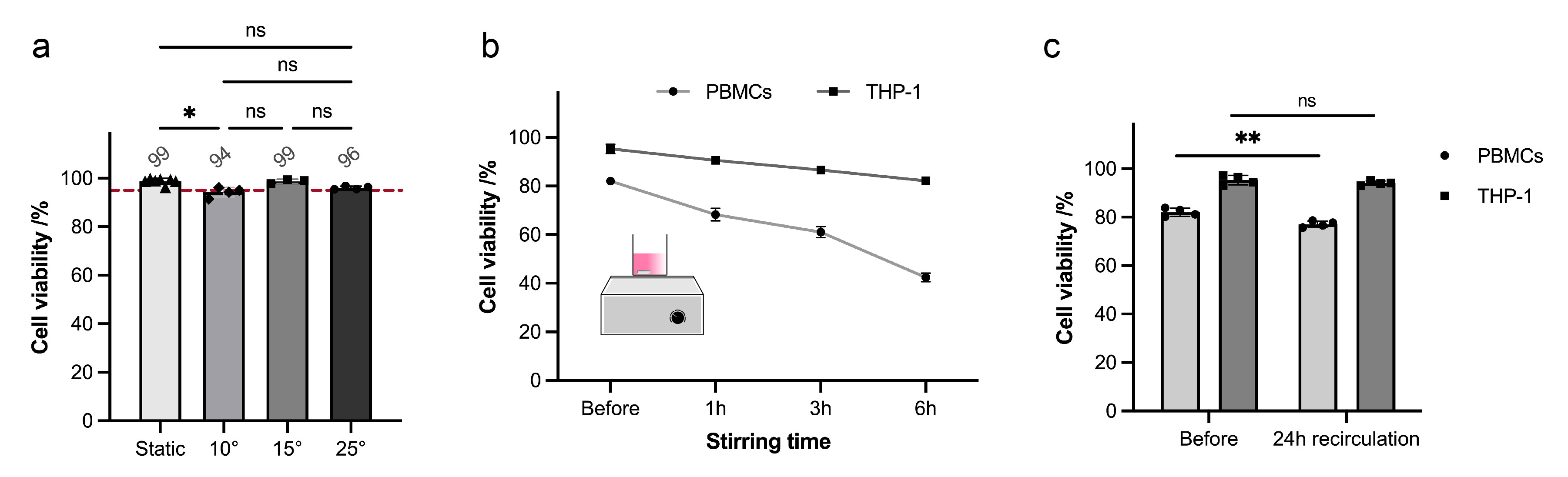
**

**Figure S2.** Viability of THP-1 cells and PBMCs under static culture, magnetic stirring, or 24 h of continuous recirculation in the UniPlate. a) Viability of THP-1 cells after 24 h of static culture or 24 h of continuous recirculation in UniPlate under different tilt angle conditions. At least n=3 for each condition. Statistical significance was determined using Kruskal-Wallis one-way ANOVA on ranks with Dunn’s post hoc test. *p < 0.05, **p < 0.01, ***p < 0.001, ****p < 0.0001. “ns” indicates not statistically significant. b) Viability of THP-1 cells and PBMCs under magnetic stirring conditions at different time points. n=4 for each condition. c) Viability of THP-1 cells and PBMCs after 24 h of continuous recirculation in the UniPlate at 25° tilt angle. n=4 for each condition. Statistical significance was determined using one-way ANOVA with the Holm–Sidak method. *p < 0.05, **p < 0.01, ***p < 0.001, ****p < 0.0001. “ns” indicates not statistically significant.


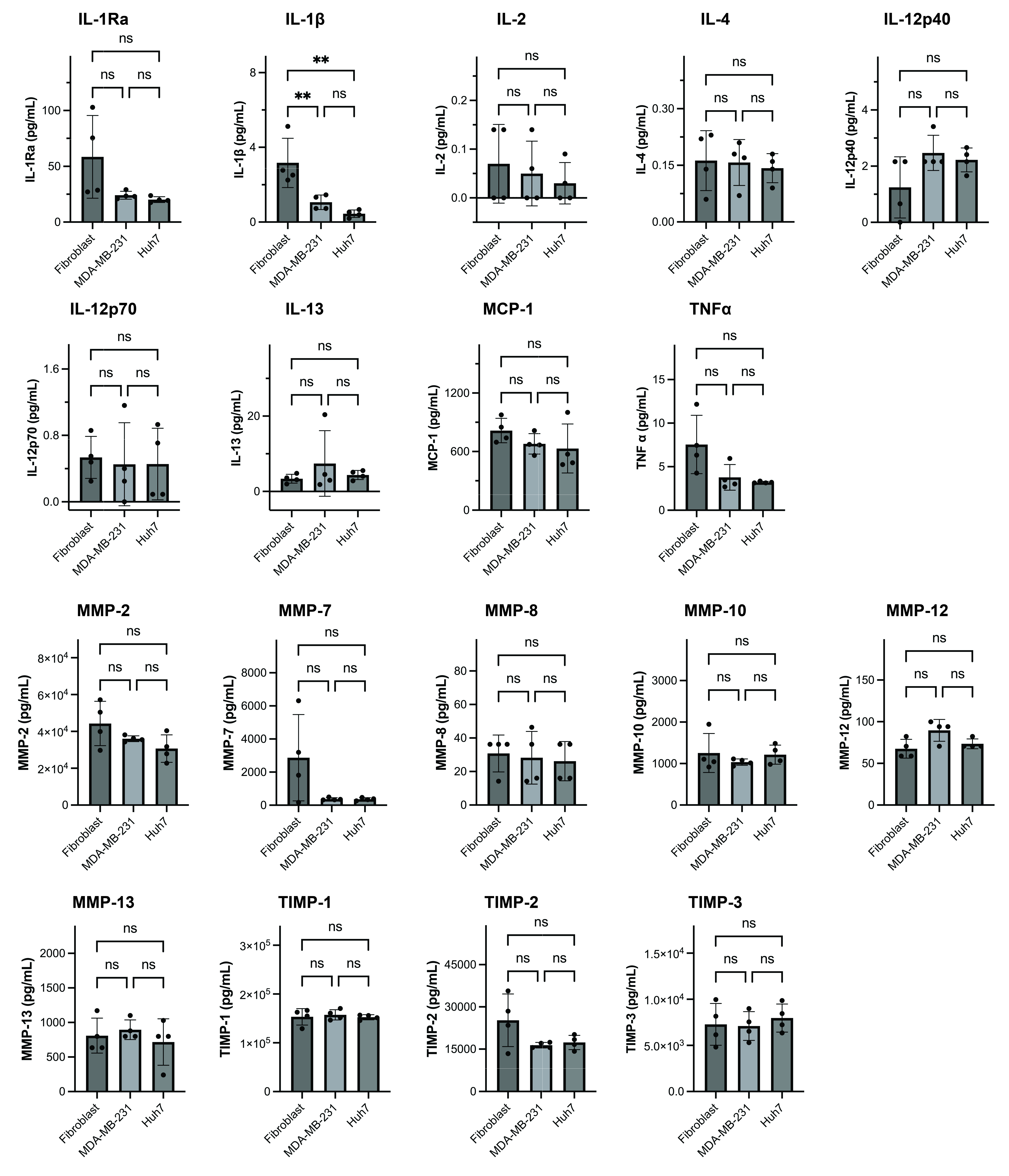


**Figure S3.** Secretion levels of all analyzed inflammatory-related cytokines, including IL-1Ra, IL-1β, IL-2, IL-4, IL-12p40, IL-12p70, IL-13, MCP-1, TNF-α, and MMPs cytokines, including MMP-2, MMP-7, MMP-8, MMP-10, MMP-12, MMP-13, TIMP-1, TIMP-2, TIMP-3 in collected media perfusates from three cancer spheroid models with fibroblast, MDA-MB-231, or Huh7 spheroids. n=4 for each condition. Statistical significance was determined using one-way ANOVA with the Holm–Sidak method or Kruskal-Wallis one-way ANOVA on ranks with Dunn’s post hoc test. *p < 0.05, **p < 0.01, ***p < 0.001, ****p < 0.0001. “ns” indicates not statistically significant.


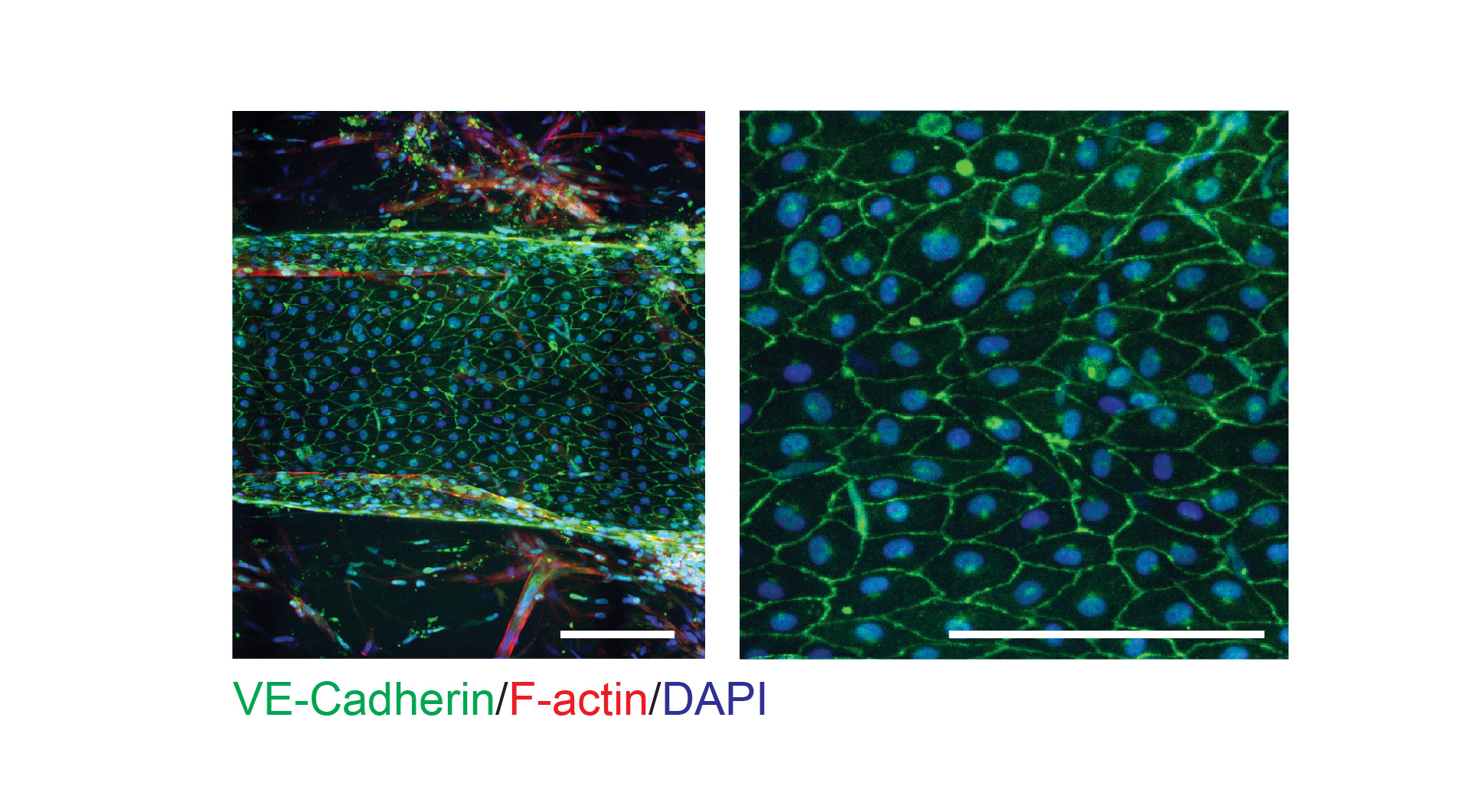


**Figure S4.** Immunofluorescent staining of a vascularized cancer spheroid tissue with MDA-MB-231 spheroids. The cells were stained with VE-Cadherin (green), F-actin (red), and DAPI (blue). Scale bar: 200 µm.
